# 5-year versus risk-category-specific screening intervals for cardiovascular disease prevention: a cohort study

**DOI:** 10.1016/S2468-2667(19)30023-4

**Published:** 2019-04-04

**Authors:** Joni V Lindbohm, Pyry N Sipilä, Nina J Mars, Jaana Pentti, Sara Ahmadi-Abhari, Eric J Brunner, Martin J Shipley, Archana Singh-Manoux, Adam G Tabak, Mika Kivimäki

**Affiliations:** a Clinicum, Department of Public Health, University of Helsinki, Helsinki, Finland; b Helsinki Institute of Life Science, University of Helsinki, Helsinki, Finland; c Institute for Molecular Medicine Finland, University of Helsinki, Helsinki, Finland; d Department of Public Health, University of Turku, Turku, Finland; e Department of Epidemiology and Public Health, University College London, London, UK; f INSERM, U1018, Centre for Research in Epidemiology and Population Health, Paris, France; g 1st Department of Medicine, Semmelweis University Faculty of Medicine, Budapest, Hungary

## Abstract

**Background:**

Clinical guidelines suggest preventive interventions such as statin therapy for individuals with a high estimated 10-year risk of major cardiovascular events. For those with a low or intermediate estimated risk, risk-factor screenings are recommended at 5-year intervals; this interval is based on expert opinion rather than on direct research evidence. Using longitudinal data on the progression of cardiovascular disease risk over time, we compared different screening intervals in terms of timely detection of high-risk individuals, cardiovascular events prevented, and health-care costs.

**Methods:**

We used data from participants in the British Whitehall II study (aged 40–64 years at baseline) who had repeated biomedical screenings at 5-year intervals and linked these data to electronic health records between baseline (Aug 7, 1991, to May 10, 1993) and June 30, 2015. We estimated participants' 10-year risk of a major cardiovascular event (myocardial infarction, cardiac death, and fatal or non-fatal stroke) using the revised Atherosclerotic Cardiovascular Disease (ASCVD) calculator. We used multistate Markov modelling to estimate optimum screening intervals on the basis of progression rates from low-risk and intermediate-risk categories to the high-risk category (ie, ≥7·5% 10-year risk of a major cardiovascular event). Our assessment criteria included person-years spent in a high-risk category before detection, the number of major cardiovascular events prevented and quality-adjusted life-years (QALYs) gained, and screening costs.

**Findings:**

Of 6964 participants (mean age 50·0 years [SD 6·0] at baseline) with 152 700 person-years of follow-up (mean follow-up 22·0 years [SD 5·0]), 1686 participants progressed to the high-risk category and 617 had a major cardiovascular event. With the 5-year screening intervals, participants spent 7866 (95% CI 7130–8658) person-years unrecognised in the high-risk group. For individuals in the low, intermediate-low, and intermediate-high risk categories, 21 alternative risk category-based screening intervals outperformed the 5-yearly screening protocol. Screening intervals at 7 years, 4 years, and 1 year for those in the low, intermediate-low, and intermediate-high-risk category would reduce the number of person-years spent unrecognised in the high-risk group by 62% (95% CI 57–66; 4894 person-years), reduce the number of major cardiovascular events by 8% (7–9; 49 events), and raise 44 QALYs (40–49) for the study population.

**Interpretation:**

In terms of timely preventive interventions, the 5-year screening intervals were unnecessarily frequent for low-risk individuals and insufficiently frequent for intermediate-risk individuals. Screening intervals based on risk-category-specific progression rates would perform better in terms of preventing major cardiovascular disease events and improving cost-effectiveness.

**Funding:**

Medical Research Council, British Heart Association, National Institutes on Aging, NordForsk, Academy of Finland.

## Introduction

Many national and international guidelines for primary prevention of cardiovascular disease recommend the estimated risk of a major cardiovascular event as the best guide to intervention decisions.^1^, ^2^, ^3^, ^4^, ^5^, ^6^ The 2016 European guidelines suggest screening every 5 years for men older than 40 years and women older than 50 years,^2^ whereas the 2013 American Heart Association/American College of Cardiologists (AHA/ACC) guidelines^5^ recommend screening at 4–6-year intervals for individuals without cardiovascular disease whose 10-year risk of a major cardiovascular event is lower than 7·5%, as calculated with the Atherosclerotic Cardiovascular Disease (ASCVD) algorithm. After the risk reaches 7·5%, the AHA/ACC guidelines^5^ classify individuals as being at high risk and recommend implementation of preventive interventions and more frequent risk-factor assessments. A 2018 New Zealand Guideline recommends risk-category-specific 10-year, 5-year, and 2-year screening intervals for low-risk, intermediate-low-risk, and intermediate-high-risk individuals for a composite outcome of hospitalisation or death from ischaemic heart disease (including unstable angina), stroke, transient ischaemic attack, heart failure, or peripheral vascular disease.^1^ However, none of these screening intervals are based on observed cardiovascular disease risk progression. Instead, they stem mainly from expert opinion.^6^

**Research in context**

**Evidence before this study**

Screening for cardiovascular disease risk factors allows detection of high-risk individuals and determination of initiation of preventive interventions such as statin therapy, but optimal screening intervals for people with low or intermediate cardiovascular risk remain unclear. We searched PubMed for current cardiovascular disease risk-estimation guidelines and their screening recommendations on Sept 30, 2018, using the Medical Subject Headings search terms “myocardial infarct”, “stroke”, “guideline”, and “risk assessment”. Many primary prevention guidelines recommend use of estimated 10-year risk of major cardiovascular events to guide initiation of interventions and screening decisions. The most common recommendation was 5-yearly screening for all individuals at low or intermediate risk, although other screening intervals, such as 10-yearly and 2-yearly screenings, were also recommended. These intervals were expert recommendations rather than based on evidence from observed cardiovascular disease risk progression.

**Added value of this study**

According to this observational study using repeat data from 6964 middle-aged British men and women, the average progression time from the categories of low or intermediate risk of a major cardiovascular event to the high-risk category (ie, ≥7·5% 10-year risk of a major cardiovascular event) depends on the initial risk category. In terms of timely initiation of intensified preventive interventions when the high-risk category is achieved, 5-yearly screening was unnecessarily frequent for low-risk individuals and insufficiently frequent for intermediate-risk individuals. We estimate that, on the basis of observed progression of major cardiovascular event risk, risk-category-specific screening intervals are more optimum than is the 5-yearly interval. For example, 7-year, 4-year, and 1-year screening intervals for low-risk, intermediate-low-risk, and intermediate-high-risk individuals would reduce the number of person-years spent unrecognised in the high-risk group and prevent major cardiovascular events without increasing costs. In terms of major cardiovascular events prevented and quality-adjusted life-years gained, 21 other screening intervals based on initial estimated risk category were also better than the uniform 5-yearly screening for everyone.

**Implications of all the available evidence**

Compared with the uniform 5-yearly screening strategy, screening intervals based on risk category were estimated to prevent a larger proportion of major cardiovascular events, to increase quality-adjusted life-years, and to reduce health-care costs. These findings suggest that one size fits all is not an optimum screening approach and support a change from uniform screening intervals to ones that are dependent on the estimated level of risk. Further research is necessary to assess the generalisability of our findings and to evaluate the benefits and costs in relation to lifetime risk rather than to 10-year estimated risk.

The 2015 PESA study^7^ revealed substantial differences in subclinical atherosclerosis among individuals with estimated risk of major cardiovascular events lower than 7·5% according to the ASCVD algorithm. General subclinical atherosclerosis occurred at four to six sites in 8% of participants whose estimated risk was lower than 5·0%, and in 24% of those whose estimated risk was 5·0–7·5%. Only 44% and 21% of individuals, respectively, in these two categories were completely free of atherosclerosis. Because the degree of atherosclerosis varies among individuals below the ASCVD 7·5% risk threshold, a uniform 5-year screening interval might not be optimal for everyone in the low-risk and intermediate-risk categories. However, evidence for the 10-year progression of risk of a major cardiovascular event at the population level in those risk categories remains scarce.^2^, ^4^, ^5^

In this prospective cohort study, we used a revised version of the ASCVD algorithm to estimate distributions of progression times from low-risk and intermediate-risk categories to the high-risk category of a major cardiovascular event (defined as myocardial infarction, death from coronary heart disease, or fatal or non-fatal stroke). The high-risk category is the threshold for considering preventive medication such as statin therapy. On the basis of these estimates, we sought to establish an optimal screening protocol for low-risk and intermediate-risk individuals and compared it with the current recommendation of 5-yearly screenings^2^, ^4^, ^5^ in terms of timely detection of high-risk individuals, major cardiovascular events prevented, quality-adjusted life-years (QALYs) gained, and costs.

## Methods

### Study design and participants

We used data from the British Whitehall II cohort study.^8^ In 1985, all civil servants aged 35–55 years and working in 20 government departments in London, UK, were invited by letter to participate; 10 308 (73%) of 14 121 agreed. The clinical examination at study entry between Sept 10, 1985, and March 29, 1988, did not include all cardiovascular risk factors. Participants underwent clinical examinations for a comprehensive set of risk factors in line with European, British, and US guidelines at 5-year intervals^2^, ^4^, ^5^ between Aug 7, 1991, and May 10, 1993; April 24, 1997, and Jan 8, 1999; Oct 8, 2002, and Sept 10, 2004; Oct 10, 2007, and Nov 18, 2009; and Jan 27, 2012, and Oct 30, 2013.

The baseline for this study was Aug 7, 1991, to May 10, 1993 (the first comprehensive risk factor screening), and participants were eligible for the present analysis if they had participated in at least two risk-factor screenings between this period and Jan 27, 2012, to Oct 30, 2013, or had participated in one screening and had a major cardiovascular event or died during follow-up. We excluded people with evidence of stroke, myocardial infarction, heart failure, atrial fibrillation, coronary artery bypass graft, or percutaneous coronary intervention at baseline.

At each examination, participants provided written informed consent for inclusion. Research ethics approval was granted by the University College London Hospital Committee on the Ethics of Human Research.

### Procedures

Data on age and smoking were collected with standard self-administered questionnaires. Experienced clinical nurses measured height, weight, and systolic and diastolic blood pressure, and took blood samples for lipid and glucose measurements.^8^ Diabetes was defined as a fasting glucose concentration of at least 7 mmol/L or use of an antidiabetic drug. Additionally, participants brought all their medication to the clinical examination and thus provided information on their use of statins, anticoagulants, and antihypertensives.

We calculated the ASCVD estimator recommended by the AHA/ACC guidelines on the basis of the following variables: age, sex, total cholesterol, HDL cholesterol, systolic blood pressure, antihypertensive medication (yes or no), smoking (yes or no), and diabetes (yes or no).^5^

Major cardiovascular events were defined as fatal coronary heart disease, non-fatal myocardial infarction, and fatal or non-fatal stroke. 10 297 (>99%) of 10 308 participants in the British Whitehall II cohort study were traced successfully and have been followed up for mortality through the national mortality register kept by the National Health Service (NHS) Central Registry, which provides information on the date and cause of each death. Coronary heart disease mortality was defined by International Classification of Diseases (ICD)-9 codes 410–414 or ICD-10 codes I20–25 and stroke mortality by ICD-9 codes 430–438 or ICD-10 codes I60–69.

Non-fatal myocardial infarction was defined following the WHO multinational monitoring of trends and determinants in cardiovascular disease (MONICA) criteria based on British Whitehall II cohort study electrocardiograms, hospital acute electrocardiograms, and cardiac enzymes, and was validated by means of discharge diagnoses from NHS Hospital Episode Statistics (HES; ICD-10 code I21) data or general practitioner confirmation.^9^ Classification was done independently by two trained coders (Whitehall II personnel), with adjudication in the event of disagreement.

Ascertainment for non-fatal stroke was based on self-reported diagnosis and use of the MONICA-Augsburg stroke questionnaires that capture symptoms associated with events, even if the participant did not report having had a diagnosis. If a participant responded positively to at least one of these questionnaires, their histories were corroborated with the general practitioner's confirmation, HES data linkage, or manual retrieval of hospital medical records reviewed by a stroke clinician.^10^ The ICD-codes used for stroke ascertainment were ICD-9 430, 431, 434, and 436, and ICD-10 I60, I61, I63, and I64.

Participants were followed up until incident myocardial infarction, stroke, death, or June 30, 2015 (the date on which cleared outcome data on major cardiovascular events were available), whichever came first. In previous validation analyses, the estimates of associations between cardiovascular risk factors and major cardiovascular events were similar for MONICA and electronic health-record-based ascertainment methods.^9^

### Statistical analysis

First, we assessed whether the ASCVD calculator showed appropriate discrimination and absolute risk estimation in this population. The algorithm showed good discrimination, but it overestimated the absolute risk in this population (appendix pp 1–6).^5^ This overestimation is expected because prevalence of risk factors, access to and compliance with treatments, and incidence of cardiovascular disease occur at more favourable levels in occupational cohorts relative to the general population (known as the healthy-worker effect).^11^ We therefore revised the ASCVD calculator and used this revised version to estimate the 10-year risk of a major cardiovascular event at each of the five clinical screenings. In the revision, we used the Cox proportional hazards model,^12^ the same variables, and the same protocol that were used in derivation of the original ASCVD calculator.^5^ After this revision, the calculator did not overestimate 10-year risk of major cardiovascular disease events and had Harrell's *C* index of 0·72 (appendix pp 1–6).

We used multistate Markov models to model risk progression across the revised ASCVD risk categories. Multistate Markov models, which are useful for modelling progression of chronic disease risk in medical research,^13^, ^14^, ^15^, ^16^, ^17^ are based on data from repeated individual-level measurements and from a transition matrix that estimates changes, both adverse and favourable, in risk category.^18^ These models allow the estimation of both progression and recovery in terms of risk categories and can be used to estimate the probability of change from one risk state to another and the percentage of individuals in each risk category after a certain time interval. A further useful feature of Markov models is that they allow outcomes, such as major cardiovascular events, and competing outcomes, such as death, that would end the follow-up at any time, to be incorporated into the modelling.

We used R package *msm*^18^ to model risk progression across risk categories (states in the multistate model), as defined by revised 10-year ASCVD-estimated risk of a major cardiovascular event: low (0 to <2·5%), intermediate-low (2·5 to <5·0%), intermediate-high (5·0 to <7·5%), and high risk (≥7·5%). We chose these categories to represent progression towards the high-risk (7·5%) major cardiovascular event threshold at which consideration of treatment is recommended according to AHA/ACC guidelines.^5^

We modelled risk progression as snapshots in time, allowing both forward and backward transitions between risk categories over time, and we treated major cardiovascular events as an absorbing category (ie, no progression estimated thereafter) and death as a competing absorbing category. A transition intensity matrix derived from the multistate model provided transition probabilities and the average time spent in each category. In multistate models, we used the quasi Newton Broyden–Fletcher–Goldfarb–Shanno algorithm for optimisation^18^ and achieved the 1 × 10^−16^ convergence criteria. To evaluate convergence to anything other than maximum likelihood, we ran our multistate model with variations in initial transition values, but the results were robust and remained unchanged (appendix pp 6–8). We did this assessment because in some cases the results of the multistate model can be sensitive to the choice of initial transition values.^18^

To derive optimal screening intervals, we defined the shortest possible screening interval to 1 year, which is used for high-risk individuals,^5^ but did not limit the longest possible screening interval. Because the AHA/ACC guideline recommends a more frequent screening interval for high-risk individuals,^5^ we did not allow backward transfer from high-risk category in the final model when deriving the optimal screening intervals. However, we allowed backward transition to show observed risk reduction, and in this sensitivity analysis the progression-time estimates did not change substantially. Comparison of the observed and ASCVD-estimated numbers of individuals in each risk category showed that the multistate model fitted our data well (appendix pp 6–8). Additionally, the 10-year major cardiovascular event risk estimates derived from the multistate model and the revised ASCVD calculator using Cox regression were similar (appendix p 9).

We estimated the costs for an NHS health check as £5·11 per invitation and £13·28 per screening^19^ and assumed that successful statin treatment would save 0·01 major cardiovascular events per person-year.^20^ We derived the cost-effectiveness and QALYs gained by statin treatment from the West of Scotland Coronary Prevention Study (WOSCOPS),^21^ in which participants were randomly assigned to primary prevention with pravastatin or placebo, and comprehensive real-world and long-term follow-up data on all cardiovascular outcomes and all related costs were collected. We estimated the screening costs and QALYs gained per year for the population of England and Wales aged between 40 and 64 years (the same age range as in our cohort at baseline) by using the population estimates derived from the Office for National Statistics^22^ and 10-year risk distributions for major cardiovascular events (estimated with the QRISK2 calculator) derived from the primary care population of England and Wales.^23^ The estimates of QALYs and costs reduced with prevention of major cardiovascular events are discounted with an annual discount factor of 3·5% as recommended by the UK treasury,^21^ whereas the screening costs were not discounted to avoid overestimation of benefits.

To explore whether selection bias might have occurred due to missing data, we undertook a sensitivity analysis in which we used multiple imputation with chained equations based on the Nelson-Aalen estimator, on outcome data, on ethnic origin (white or non-white), and on repeated measurements of observed 10-year risk, socioeconomic status, alcohol consumption, physical activity, body-mass index, dietary approaches to stop hypertension (DASH) diet score, family history of myocardial infarction or stroke (in either parent or any sibling), and a general health questionnaire (with 30 questions).

We imputed the data in wide form to take into account the clustering of repeated measurements within individuals. The diagnostics of our imputation model suggested that 10 iterations and 25 imputations were sufficient to produce reproducible results (appendix pp 14–16). The 25 datasets produced from imputations were then analysed separately with the multistate Markov models, and the results were combined using Rubin's rules.^24^ This procedure takes into account the uncertainty in the imputation as well as uncertainty due to random variation. In these analyses, we examined whether the association of the revised ASCVD-estimated risk categories with incident major cardiovascular events in the imputed datasets (that aim to control potential selection bias) differed from the association in our main analysis that included missing data. All analyses were done with Stata version SE 14.2 and R version 3.4.3.

### Role of the funding source

The funders of the study had no role in study design, data collection, data analysis, data interpretation, or writing of this report. JVL and MK had full access to all the data in the study and had final responsibility for the decision to submit for publication.

## Results

**Figure 1:**
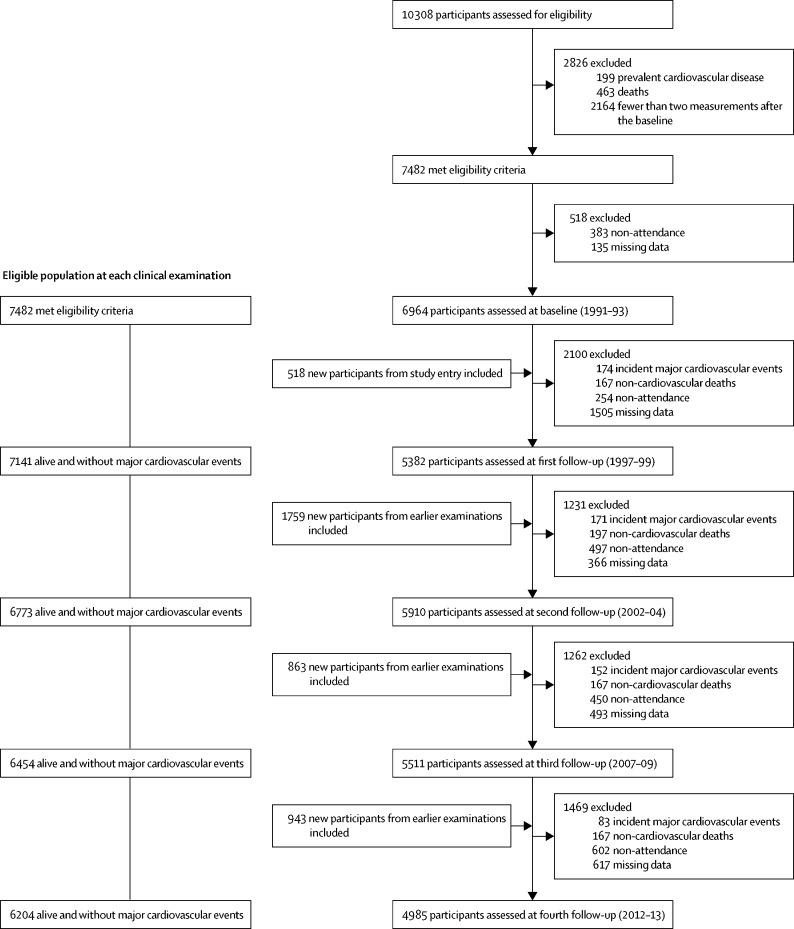
Flow chart of sample selection at each clinical examination

Our baseline cohort comprised 6964 participants (4866 men and 2098 women; figure 1) and provided 152 700 person-years of follow-up. Mean age of the participants at baseline was 50·0 years (SD 6·0; table 1). The appendix (p 11) provides age distributions in each risk category by clinical examination. Antihypertensive, lipid-lowering, and anticoagulation medication use increased over the follow-up period, which is consistent with the increasing percentage of high-risk individuals over time (appendix p 6).

**Table 1:** Characteristics of the study population at baseline and at the last screening

		**All (n=6964)**	**Men (n=4866)**	**Women (n=2098)**
**Baseline examination**				
Age, years		50·0 (6·0)	49·8 (6·0)	50·5 (6·1)
Systolic blood pressure, mm Hg		120·4 (13·5)	121·7 (13·1)	117·4 (13·9)
Total cholesterol, mmol/L		6·5 (1·1)	6·5 (1·1)	6·5 (1·2)
HDL cholesterol, mmol/L		1·4 (0·4)	1·3 (0·4)	1·7 (0·4)
Diabetes		137 (2%)	97 (2%)	40 (2%)
Current smoker		939 (13%)	601 (12%)	338 (16%)
Antihypertensive treatment		450 (6%)	265 (5%)	185 (9%)
**10-year risk category at baseline**				
Original ASCVD				
	<2·5% (low)	2612 (38%)	1174 (24%)	1438 (69%)
	2·5% to <5% (intermediate-low)	1857 (27%)	1403 (29%)	454 (22%)
	5% to <7·5% (intermediate-high)	1024 (15%)	894 (18%)	130 (6%)
	≥7·5% (high)	1471 (21%)	1395 (29%)	76 (4%)
Revised ASCVD				
	<2·5% (low)	3733 (54%)	2207 (45%)	1526 (73%)
	2·5% to <5% (intermediate-low)	2119 (30%)	1713 (35%)	406 (19%)
	5% to <7·5% (intermediate-high)	653 (9%)	547 (11%)	106 (5%)
	≥7·5% (high)	459 (7%)	399 (8%)	60 (3%)
**10-year risk category at fourth follow-up**				
Number of patients		4985	3581	1404
Original ASCVD				
	<2·5% (low)	51 (1%)	0	51 (4%)
	2·5% to <5% (intermediate-low)	288 (6%)	18 (1%)	270 (19%)
	5% to <7·5% (intermediate-high)	406 (8%)	163 (5%)	243 (17%)
	≥7·5% (high)	4240 (85%)	3400 (95%)	840 (60%)
Revised ASCVD				
	<2·5% (low)	314 (6%)	92 (3%)	222 (16%)
	2·5% to <5% (intermediate-low)	1377 (28%)	943 (26%)	434 (31%)
	5% to <7·5% (intermediate-high)	1149 (23%)	846 (24%)	303 (22%)
	≥7·5% (high)	2145 (43%)	1700 (47%)	445 (32%)

Data are mean (SD) or number (%). ASCVD=Atherosclerotic Cardiovascular Disease.

During a mean follow-up of 22·0 years (SD 5·0), 1686 participants progressed to the high-risk category, 617 had a major fatal or non-fatal cardiovascular event, and 788 died due to non-cardiovascular causes. The major cardiovascular events comprised 332 non-fatal myocardial infarctions, 71 coronary heart disease deaths, and 205 non-fatal and nine fatal strokes. Of 617 major cardiovascular events, 360 (58%) occurred in individuals at high risk and 257 (42%) occurred in those with low or intermediate risk at their most recent 5-year screening.

**Figure 2:**
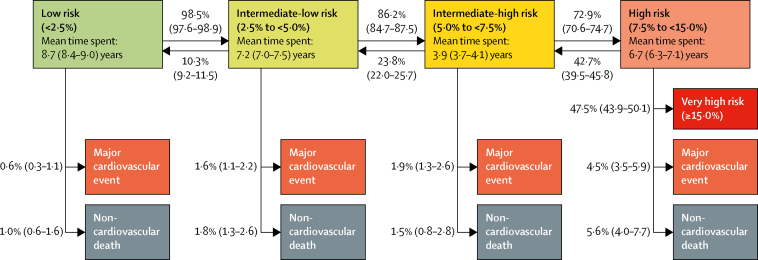
Estimated mean time spent in each major cardiovascular event 10-year-risk category and transition probabilities to the next risk category, incident major cardiovascular event (fatal or non-fatal) and non-cardiovascular death Transition probabilities between groups and to events or deaths are presented next to arrows as percentage (95% CI). Mean time spent is calculated on the basis of all transitions and includes all individuals who visited the category during follow-up. The high-risk category has been split into two: high risk (7·5 to <15·0%) and very high risk (≥15%) to describe risk progression.

Time spent in each risk category ranged from a mean of 8·7 years (95% CI 8·4–9·0) spent in the low-risk (<2·5%) category to 3·9 years (3·7–4·1) in the intermediate-high-risk (5·0 to <7·5%) category (figure 2). The mean time spent before reaching the high-risk category was 19·8 years (95% CI 19·4–20·3) for participants in the low-risk category, 11·1 years (10·7–11·5) in the intermediate-low-risk category, and 3·9 years (3·7–4·1) in the intermediate-high-risk category. Participants who reached the high-risk category spent a mean of 6·7 years (6·3–7·1) in this category, after which an estimated 42·7% (95% CI 39·5–45·8) showed a reduced risk and 47·2% (43·9–50·1) progressed to the very-high-risk category (≥15%), 4·5% (3·5–5·9) had a major cardiovascular event, and 5·6% (4·0–7·7) died from non-cardiovascular causes.

Participants from low-risk and intermediate-low-risk categories progressed towards higher risk categories more often than did those at high risk; the reverse transition was more common from the higher risk categories (figure 2). All factors in the ASCVD calculator predicted progression from a lower to a higher risk category, with the strongest predictors being smoking, diabetes, and systolic blood pressure (appendix p 12). Secular trends in risk factors suggested that individuals quitting smoking, reducing their systolic blood pressure and cholesterol, and increasing their HDL concentration contributed to transitions from a higher to a lower risk category (appendix pp 12–14).

Since the progression rate to the high-risk category varied depending on the initial risk category, the proportion of participants who progressed to the high-risk category before the next screen in the 5-year screening interval also varied depending on risk category: 133 (2%) of 6630 person observations in the low-risk, 895 (12%) of 7462 in the intermediate-low-risk, and 1588 (45%) of 3528 in the intermediate-high-risk category progressed to the high-risk category before the next screen.

**Figure 3:**
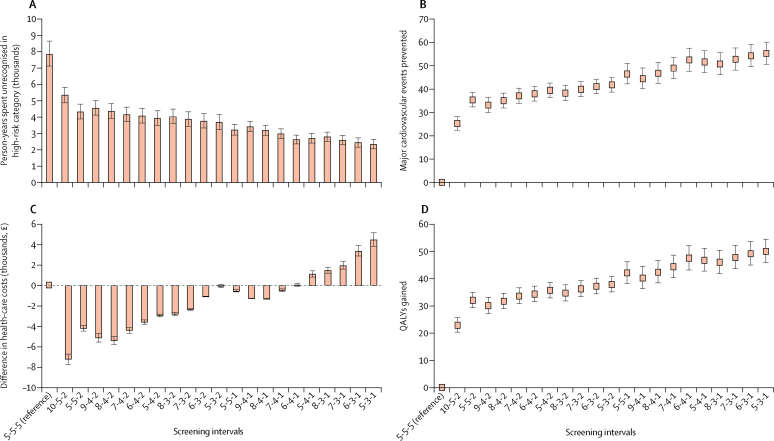
Comparison of 21 risk-category-specific screening intervals with the uniform 5-year screening interval (A) Person-years spent unrecognised in high-risk category, (B) number of major cardiovascular events prevented, (C) difference in health-care costs, and (D) QALYs gained in the study population over the time of 20 years. The screening intervals are in years for those in low-risk, intermediate-low-risk, and intermediate-high-risk categories. Data are estimates with 95% CIs. QALY=quality-adjusted life-year.

Of the 21 alternative risk-category-based screening interval protocols, 16 were associated with costs lower than or equal to those for the 5-year screening interval (figure 3). All 21 alternative models of risk-category-specific screening intervals outperformed the uniform 5-year screening interval in terms of person-years spent unrecognised in the high-risk category, QALYs gained, and major cardiovascular events prevented.

On the basis of progression rates to high risk from each risk category, we estimated the most optimal risk-category-specific screening intervals that would not elevate health-care cost but would lead to more timely detection of high-risk individuals and a greater number of major cardiovascular events prevented and QALYs gained. The protocol was 7-year, 4-year, and 1-year screening intervals for low-risk, intermediate-low-risk, and intermediate-high-risk individuals, respectively (figure 3; appendix p 10).

Compared with 5-year screenings, screenings using 7-year, 4-year, and 1-year intervals would reduce person-years spent unidentified in the high-risk category by 62% (95% CI 57–66; 4894 person-years). It would thus prevent an estimated 8% (95% CI 7–9; 49 events) of major cardiovascular events with earlier statin intervention over the 20 years of follow-up and reduce the percentage of major cardiovascular events originating in the low-risk and intermediate-risk groups from 42% to 34% (figure 3; table 2). With screening intervals at 10, 5, and 2 years for low-risk, low-intermediate-risk, and high-intermediate-risk categories, the corresponding estimated reductions were 32% (29–36; 2527 person-years) and 4% (3–5; 25 major cardiovascular events), respectively. The percentage of major cardiovascular events originating from low-risk and intermediate-risk groups would reduce from 42% to 38%.

**Table 2:** Comparison of two risk-category-specific screening intervals with the uniform 5-year screening interval

		5-5-5 screening interval*	10-5-2 screening interval*	7-4-1 screening interval*
Number of people at low or intermediate risk at baseline		6545	6545	6545
Number of person-years spent unidentified in high-risk category		7866 (7130 to 8658)	5339 (4885 to 5827)	2973 (2681 to 3292)
Difference in person-years spent unidentified in high-risk category		0 (reference)	−2527 (−2831 to −2245)	−4894 (−5366 to −4449)
Number of major cardiovascular events prevented		0 (reference)	25 (22 to 28)	49 (44 to 54)
Number of adverse events caused across the study period				
	Diabetes	0 (reference)	3·79 (3·37 to 4·25)	7·34 (6·67 to 8·05)
	Haemorrhagic stroke	0 (reference)	0·38 (0·34 to 0·42)	0·73 (0·67 to 0·80)
	Myopathy	0 (reference)	0·25 (0·22 to 0·28)	0·49 (0·44 to 0·54)
Number of QALYs gained		0 (reference)	23 (20 to 26)	44 (40 to 49)
Costs (thousands, £)†				
	Health-check costs	437 (428 to 445)	412 (400 to 425)	658 (627 to 693)
	Costs saved owing to earlier prevention with statin	0 (reference)	−120 (−134 to −106)	−232 (−254 to −211)
	Total costs (health-check costs − savings)	437 (428 to 445)	292 (291 to 294)	427 (416 to 439)
	Total costs compared with 5-5-5 screening interval	0 (reference)	−144 (−154 to −134)	−10 (−12 to −7)

Data are estimate (95% CI), unless otherwise specified. Figures are estimated for a 20-year period. QALY=quality-adjusted life-year.

* In years for low-risk, intermediate-low-risk, and intermediate-high-risk individuals (95% CI).

† In the Whitehall II cohort of adults aged 40–64 years at baseline. Cost of £18·39 per health check derived from Kypridemos and colleagues^19^ and costs and QALYs gained with preventive statin treatment (£47·33 and 0·00906 QALYs gained per person-year under statin treatment compared with placebo) derived from Collins and colleagues^20^ and from the West of Scotland Coronary Prevention Study.^21^ The number of adverse events estimated based on incidence estimates of 0·0015, 0·00015, and 0·00010 for diabetes, haemorrhagic stroke, and myopathy^20^ among those who would have received statin treatment.

Table 3 presents the estimated screening costs and person-years spent in the high-risk category with risk-category-based screening intervals when compared with 5-year screening intervals for the current population of England and Wales aged 40–64 years at baseline. After taking into account the extra measurement costs and cardiovascular-disease-related costs saved with preventive statin medication, the 7-4-1 screening protocol for individuals in England and Wales would lead to savings of approximately £1 200 000 per year (95% CI 900 000–1 400 000), 4564 QALYs (4163–4635) gained, and 5034 major cardiovascular events (4592–5503) prevented per year compared with results with standard 5-year screening intervals. The benefits for the 10-5-2-year screening would be approximately £15 100 000 (95% CI 14 200 000–16 100 000) in cost savings, 2475 QALYs (2219–2750) gained, and 2730 major cardiovascular events (2447–3033) prevented per year. Because of more person-years spent under statin treatment, both the 7-4-1 and 10-5-2 screening protocols would be associated with a higher number of statin-related adverse events than would the 5-yearly screening (table 3). However, compared with the reduction in major cardiovascular events, the number of adverse events would be small (table 3).

**Table 3:** Comparison of two risk-category-specific screening intervals with the uniform 5-year screening interval

		5-5-5 screening interval*	10-5-2 screening interval*	7-4-1 screening interval*
Number of people at low or intermediate risk at baseline (per 1 000 000)		12·4	12·4	12·4
Number of person-years spent unidentified in high-risk category (per 1000)		785 (713 to 863)	512 (469 to 559)	282 (254 to 313)
Difference in person-years spent unidentified in high-risk category (per 1000)		0 (reference)	−273 (−303 to −245)	−503 (−550 to −495)
Number of major cardiovascular events prevented		0 (reference)	2730 (2447 to 3033)	5034 (4592 to 5503)
Number of adverse events				
	Diabetes	0 (reference)	410 (367 to 455)	755 (689 to 825)
	Haemorrhagic stroke	0 (reference)	41 (37 to 46)	76 (69 to 83)
	Myopathy	0 (reference)	27 (24 to 30)	50 (46 to 55)
Number of QALYs gained		0 (reference)	2475 (2219 to 2750)	4564 (4163 to 4635)
Costs (millions, £)†				
	Health-check costs	41·3 (40·5 to 42·1)	39·1 (37·9 to 40·3)	63·9 (60·8 to 67·2)
	Costs saved due to earlier prevention with statin	0 (reference)	−12·9 (−14·4 to −11·6)	−23·8 (−26·0 to −21·7)
	Total costs (health-check costs − savings)	41·3 (40·5 to 42·1)	26·2 (26·0 to 26·3)	40·0 (39·1 to 41·2)
	Total costs compared with 5-5-5 screening interval	0 (reference)	−15·1 (−16·1 to −14·2)	−1·2 (−1·4 to −0·9)

Figures are estimated for 1 year. QALY=quality-adjusted life-year.

* In years for low-risk, intermediate-low-risk, and intermediate-high-risk individuals (95% CI).

† In the population of England and Wales aged 40–64 years.^22^ Cost of £18·39 per health check derived from Kypridemos and colleagues^19^ and costs and QALYs gained with preventive statin treatment (£47·33 and 0·00906 QALYs gained per person-year under statin treatment compared with placebo) derived from Collins and colleagues^20^ and from the West of Scotland Coronary Prevention Study.^21^ The number of adverse events based on incidence estimates of 0·0015, 0·00015, and 0·00010 for diabetes, haemorrhagic stroke, and myopathy^20^ among those who would have received statin treatment.

Our sensitivity analysis with imputed missing data provided similar associations to those from our main analysis that used complete cases only (appendix pp 14–16).

## Discussion

In this prospective longitudinal study, we found that risk progression accelerated with higher risk levels, suggesting that uniform 5-year screening intervals for low-risk, intermediate-low-risk, and intermediate-high-risk categories (as defined according to a revised ASCVD calculator) leads to unnecessarily long delays in detection of high-risk individuals. Compared with the uniform 5-year intervals, estimated risk-category-specific screening intervals—such as 7-year, 4-year, and 1-year screenings for low-risk, intermediate-low-risk, and intermediate-high-risk individuals, respectively—were estimated to reduce time spent unidentified in the high-risk category, to prevent major cardiovascular disease events, and to increase QALYs by more timely preventive interventions. Moreover, our analyses suggest that many protocols with screening intervals that are risk-category-specific would also reduce health-care costs in England and Wales.

We compared 21 protocols of risk-category-specific screening intervals with the uniform 5-year screening interval, and our findings provide strong support for the benefits of using risk-category-specific screening intervals. In terms of QALYs gained and major cardiovascular events prevented, all 21 alternative models of risk-category-specific screening intervals outperformed the 5-year-interval screening; 16 of them were also either less costly or the estimated costs did not exceed those related to the 5-year screening interval. The lowest estimated costs related to 10-year, 5-year, and 2-year; 8-year, 4-year, and 2-year; and 9-year, 4-year, and 2-year screening intervals for low-risk, intermediate-low-risk, and intermediate-high-risk individuals.

Few previous studies have examined cardiovascular disease risk progression. Analysis of data from the pre-statin era derived from the Tokyo Health Check-up study and the Framingham study found that among those at low risk (as defined by the Framingham General Cardiovascular Disease Risk Functions), 9% proceeded to the high-risk category within 8 years,^25^ which is similar to our results (appendix p 9). The authors concluded that rescreening should be based on baseline risk.^25^ Our study complements these findings by providing specific screening intervals that stem from observed risk progression and from comparison of different screening strategies. Additionally, our study supports earlier study findings that suggested more frequent risk factor screenings for individuals with higher levels of total cholesterol, high systolic blood pressure, or elevated glycated haemoglobin.^26^, ^27^, ^28^

The performance of the revised ASCVD calculator in the present study (*C* = 0·72) was equally good as in other major studies (*C* = 0·68–0·82).^23^, ^29^ Despite the high discrimination of the cardiovascular risk algorithms, their detection rate is modest.^30^ Thus, in our study, 42% of all major cardiovascular disease events occurred among individuals at low or intermediate risk based on their latest screen in the 5-year screening-interval strategy. The corresponding proportion was 34% with the 7-4-1-year screening strategy. This finding calls for further research to identify new biomarkers that would allow development of scalable screening instruments with a significantly higher detection ratio combined with a low false-positive rate.

The strengths of this study include its long follow-up and frequent risk-factor measurements according to the 5-year-interval guidelines, which enabled detailed modelling of 10-year progression of major cardiovascular-event risk in an era of modern preventive medicine.

The present study also has several limitations. Because our findings are from an occupational cohort with a higher proportion of men than women, they might not apply to the general population, including those not in paid employment and to groups that are more gender equal. The progression of major cardiovascular event risk in the general population might be faster than observed in our cohort, indicating that more frequent screening might prove optimal in such populations. To confirm this theory and to assess the generalisability of our findings, further large-scale research across different populations and health-care systems is essential.

Despite a high response to the survey at the successive data-collection phases, loss to follow-up accumulated over the extended time period. In our study, the results in the main analyses that included missing data and in those based on multiple-imputation datasets were similar. Multiple imputation assumes that missing values can be predicted reasonably accurately with variables included in the imputation model, and this is thought to justify the missing-at-random assumption. Our imputation model included repeated measurements of variables that related to missing values and provided results similar to those of the complete case analysis, thus indicating that major bias due to missing data and non-attendance to follow-up examinations is unlikely.

Our estimates of the health benefits from statin therapy were based on a microsimulation that took into account the effects of low compliance and on the WOSCOPS,^21^ a randomised controlled trial with a higher compliance than usually seen in real life; as such, the effects of poor compliance on screening intervals were only partially addressed in our study and these are a potential source of overestimation of the benefits of early drug therapies. Conversely, we did not consider the additional benefits of an earlier start of antihypertensive medication (because this medication was part of the risk-score algorithm) or of other intensified prevention for high-risk patients, such as more professional assistance with lifestyle changes and psychosocial risks and a tighter control of obesity and dysglycaemia.^2^ This factor might have contributed to an underestimation of the benefits of earlier detection of a high-risk state.

A further issue affecting cost-effectiveness of the screening intervals, but one impossible to cover in our analyses, is that the acceptance and application of new guidelines into practice is likely to vary depending on the settings in which they are implemented.^31^ Ultimately, trials directly comparing the cost-effectiveness of different models of screening intervals in various health-care systems would address this question.

In conclusion, this study suggests that a shift from 5-year-interval screening protocols to risk-category-specific screening intervals for low-risk, intermediate-low-risk, and intermediate-high-risk individuals could reduce or delay major cardiovascular events, reduce health-care costs, and lead to an increase in QALYs gained at the population level. Future longitudinal studies should examine cardiovascular disease risk progression in other cohorts to assess the generalisability of our findings and to provide additional evidence to guide nationwide cardiovascular disease risk screening strategies. If our results are replicated, this would support a change from uniform screening intervals to ones that are dependent on estimated level of risk.

## Data sharing

Data, protocols, and other metadata of the Whitehall II study are available to the scientific community. Please refer to the Whitehall II study data sharing policy.
