# Supplementary material for: 5-year versus risk-category-specific screening intervals for cardiovascular disease prevention: a cohort study
Source: Lancet Public Health. 2019 Apr 4;4(4):e189–99. doi: 10.1016/S2468-2667(19)30023-4 (PMC6472327; doi:10.1016/S2468-2667(19)30023-4)
Supplement: Supplementary appendix [file mmc1.pdf]

THE LANCET
Public Health

Supplementary appendix

This appendix formed part of the original submission and has been peer reviewed. We post it as supplied by the authors.

Supplement to: Lindbohm JV, Sipilä PN, Mars NJ, et al. 5-year versus risk-category-specific screening intervals for cardiovascular disease prevention: a cohort study. *Lancet Public Health* 2019; **4:** e189–99.

Appendix

Statistical analysis

Our modelling was based on the following eight steps and assumptions:

STEP 1

Because the ASCVD score overestimated the risk in the Whitehall II cohorts, we revised it in order to obtain reliable risk estimates. To identify which factors of the score contributed to the overestimation, we reconstructed the revised ASCVD score step by step and used the same methods as were used in the derivation of the original ASCVD score.[1] In model derivation, we used the baseline risk factor measurements and major cardiovascular events that occurred during a 10-year follow-up. We derived the baseline 10-year risk from our cohort and omitted the interactions and power terms of the ASCVD score if they were non-significant (with $p<0.05$) because they would have worsened the calibration. The final equation for the 10-year risk was:

$$1-(\text{Baseline survival})^{e^{(Coefficient \times Value - Mean)}}$$

For men:

Baseline survival: 0.9708825

Coefficient: (ln(AGE_YEARS) * (3.149231)) + (ln(CHOLESTEROL_mg) * (1.044396)) + (ln(HDL_mg) * (-0.9008655)) + (ln(SYSTOLIC BLOOD PRESSURE) * (2.245054)) + (ANTIHYPERTENSIVE MEDICATION) * (0.7826888) + (SMOKING) * (0.7044788) + (DIABETES) * (0.3602688)

Mean value: (ln(49.68241) * (3.149231) + (ln(249.8557) * (1.044396)) + (ln(51.13489) * (-0.9008655)) + (ln(121.8954) * (2.245054)) + (0.0531474) * (0.7826888) + (0.1223144) * (0.7044788) + (0.0192235) * (0.3602688))

For women:

Baseline survival: 0.9845597

Coefficient: (ln(AGE_YEARS) * (3.63634)) + (ln(CHOLESTEROL_mg) * (0.4460168)) + (ln(HDL_mg) * (-0.6703398)) + (ln(SYSTOLIC BLOOD PRESSURE) * (3.207205)) + (ANTIHYPERTENSIVE MEDICATION) * (0.3617712) + (SMOKING) * (0.8212462) + (DIABETES) * (0.8260577)

Mean value: (ln(50.69339) * (3.63634) + (ln(251.7281) * (0.4460168)) + (ln(65.10147) * (-0.6703398)) + (ln(117.6833) * (3.207205)) + (0.087889) * (0.3617712) + (0.1593776) * (0.8212462) + (0.0185029) * (0.8260577))

This revised algorithm had a similar discrimination (eFigure 1) but superior calibration (eFigure 2) when compared to the original ASCVD score (see calibration plots below). In addition, the revised algorithm provided 10-year risk estimates similar to those of our final multistate model that used a longer follow-up and repeated risk factor measurements. The results did not change markedly when mean or 0/1 of binary variables was used in derivation of the mean risk.

eFigure 1: Distribution of estimated 10-year risk of major cardiovascular event according to the original ASCVD calculator (white bar) and the revised ASCVD calculator (green bar) at baseline (A) and 1st (B), 2nd (C), 3rd (D), and 4th (E) follow-ups.

Discriminative ability between the original ASCVD calculator (Harrell's C = 0.71) and the revised ASCVD calculator (Harrell's C = 0.72) did not differ. However, the original ASCVD calculator was less well calibrated (eFigure 2). eFigure 1 shows the proportion of participants (y-axis) by estimated 10-year risk of major cardiovascular event (x-axis) using the original ASCVD-calculator (white bars) and the revised ASCVD-calculator (green bars). The proportions at the lower end of the estimated risk distribution were higher for the revised ASCVD calculator than for the original one, suggesting that the original ASCVD calculator overestimated the 10-year risk of major cardiovascular events in the Whitehall II cohort (parts A to E).

A) 1991-1993 clinical examination (baseline for this study)

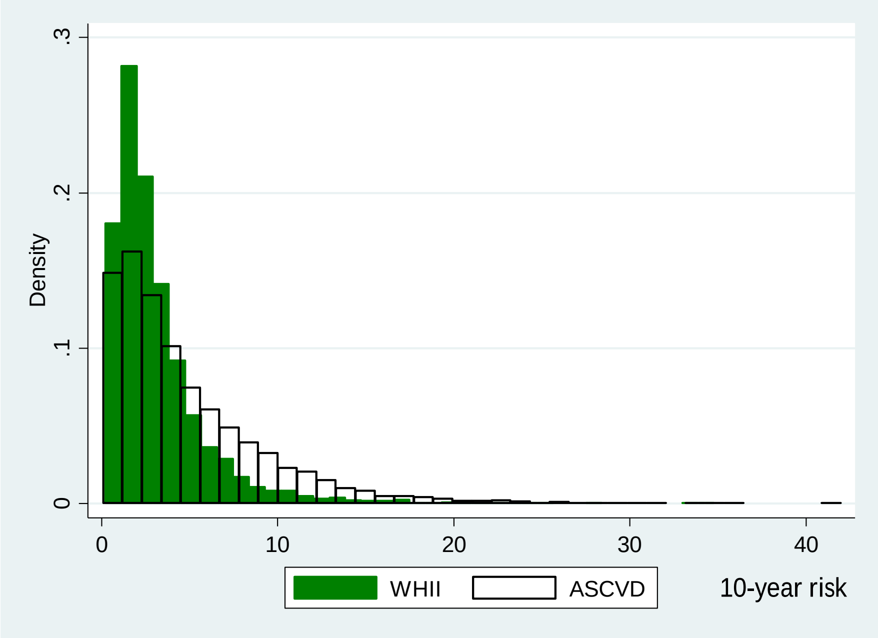

B) 1997-1999 (first follow-up)

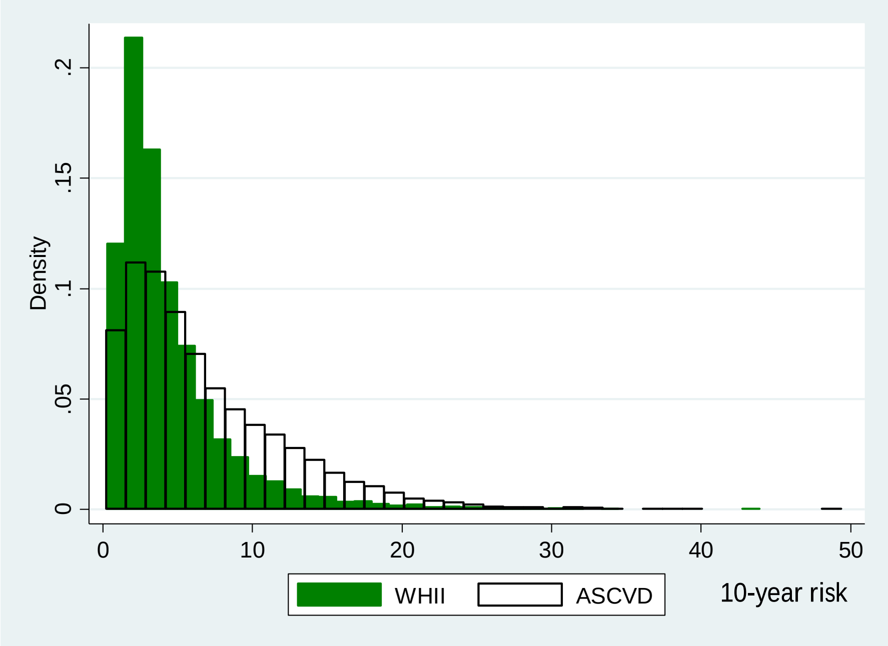

C) 2002-2004 (second follow-up)

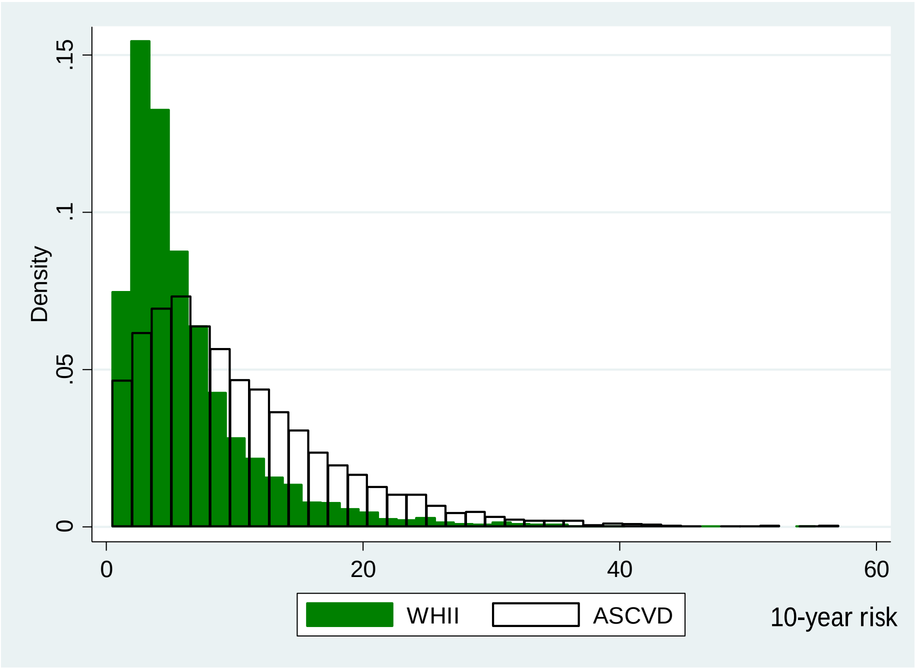

D) 2007-2009 (third follow-up)

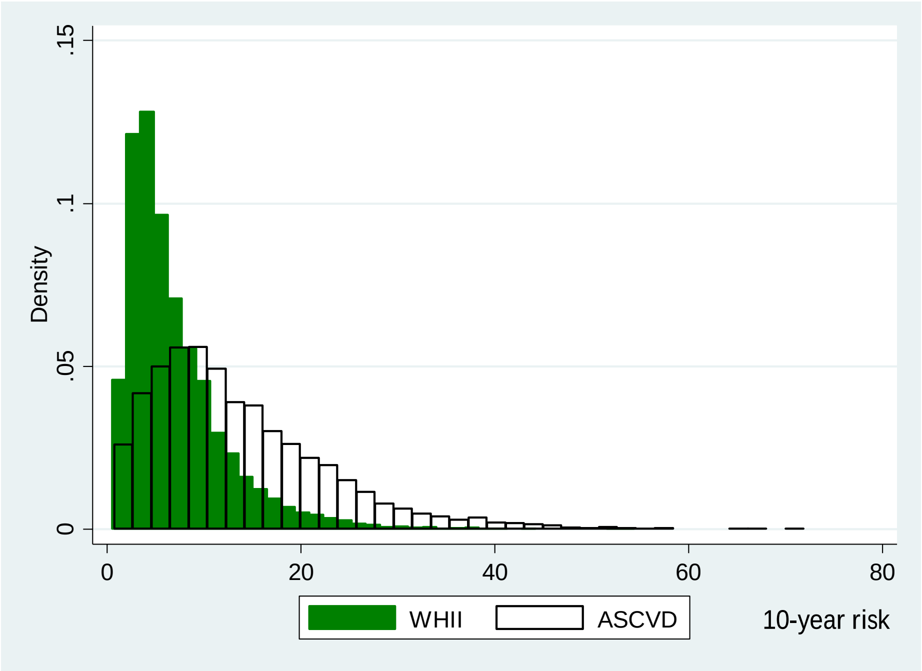

E) 2012-2013 (fourth follow-up)

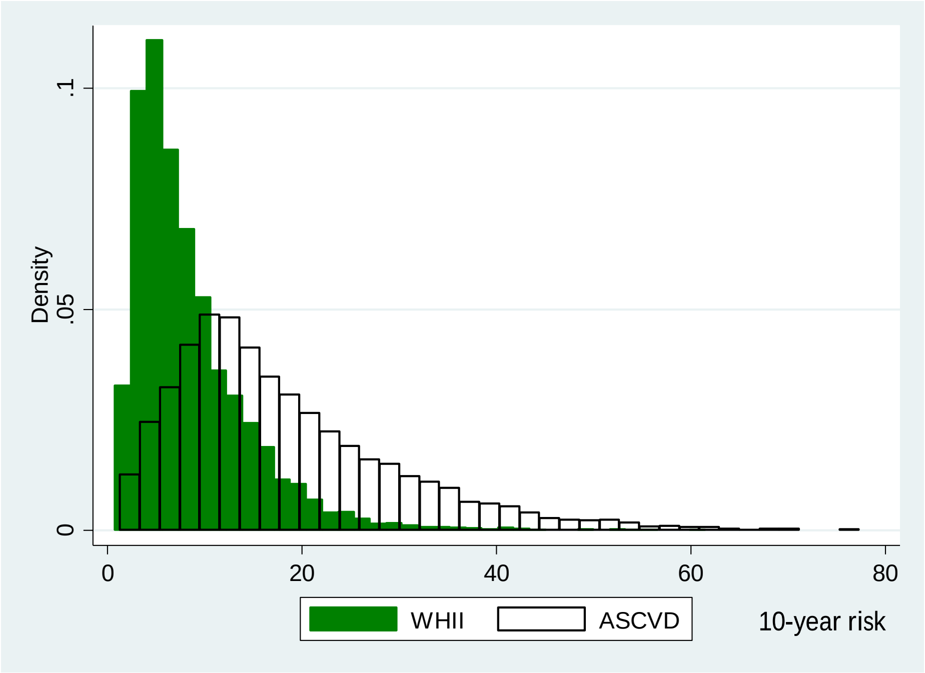

eFigure 2. Calculator performances in risk quintiles. Discrimination was assessed by Harrell's C, whereas calibration is presented with observed and expected survival curves by quintiles. Solid lines (km1-5) indicate observed and dashed lines (s1-5) predicted survival.

ASCVD calculator revised for Whitehall II cohort
Harrell´s C = 0.72

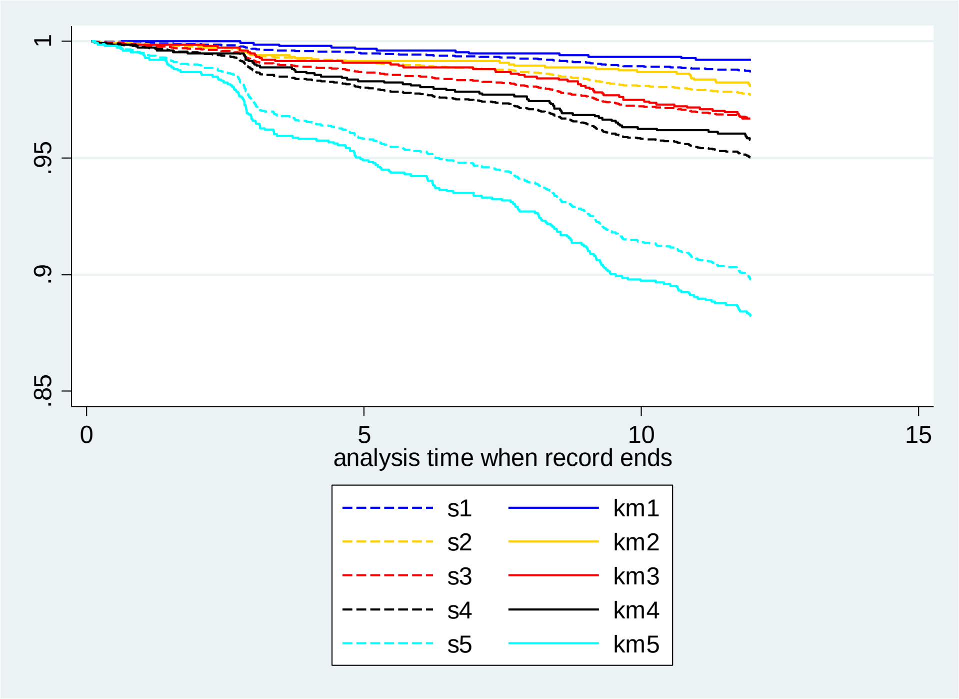

Original ASCVD calculator
Harrell´s C = 0.71

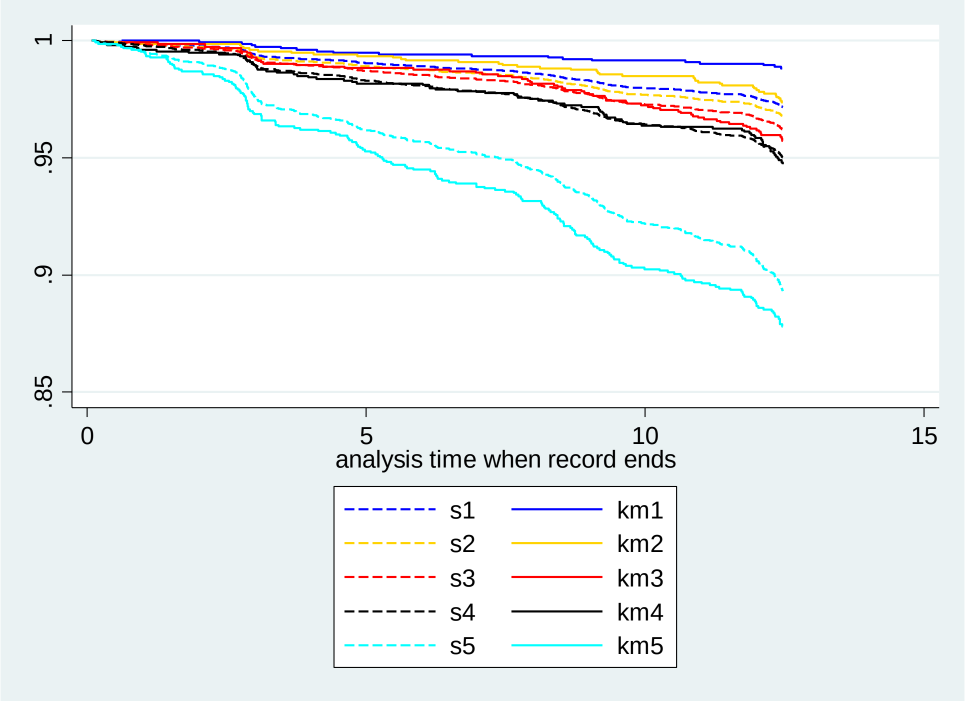

Assumptions in Step 1

- We studied non-linear associations and interactions when deriving the revised ASCVD calculator but found no strong evidence supporting these and thus did not include them in the final revised ASCVD calculator. In addition, the non-linear associations in the original ASCVD calculator led to worse calibration, so we omitted these from the final model. Proportional hazards assumption was not violated in our final model.
- We assumed that our revised ASCVD calculator would have reasonable discrimination and calibration, as was suggested by our model diagnostics.

STEP 2

We then used the revised ASCVD score to calculate participants' 10-year major cardiovascular event risk at each screening phase and divided participants into groups of low (0-2·49%), intermediate-low (2·5-4·99%), intermediate-high (5-7·49%), and high (≥7·5%) risk. We chose these categories to represent progression towards the 10-year high-risk (7·5%) threshold at which treatment is recommended according to the AHA/ACC guidelines.

Assumptions in Step 2

- We assumed that proportion of individuals in the high-risk category would correspond to those for the individuals with primary prevention medication. This assumption was confirmed, as shown in eFigure 3:

eFigure 3.
Proportion of participants using antihypertensive (blue), lipid-lowering, (orange), or anticoagulation (grey) medication (by year). Black line shows proportion of participants in the high-risk (≥7·5%) category.

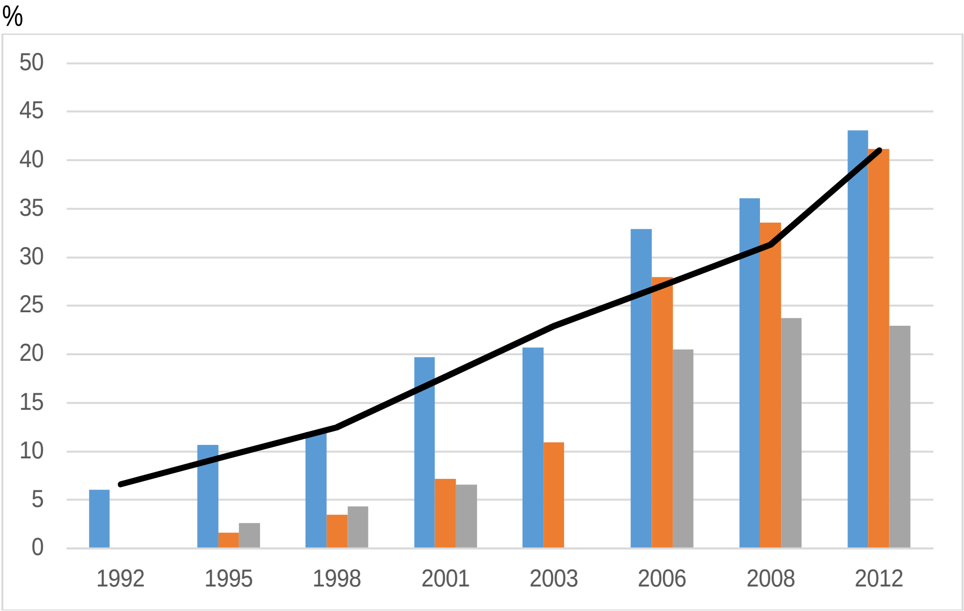

STEP 3

We then used the msm package in R[2] to construct a model describing transitions between the 10-year major cardiovascular event-risk states: low (0-2·49%), intermediate-low (2·5-4·99%), intermediate-high (5-7·49%), and high (≥7·5%) risk.

Initial values were necessary as a starting point, because the likelihood in the multistate model is maximised by numerical methods. We used crude initial transition frequency values based on those observed between state transitions and time spent in each state as recommended.[2] As reassurance that our initial values did not lead to deviation from the true maximum likelihood estimates, we changed the initial values by shortening the time spent in each state by 10% or 50%, and by lengthening it by 10% or 50% in order to simulate a real world situation in

which the actual measurement intervals differ. However, the different initial values did not change our results, suggesting that our model was robust and not affected by the initial transition frequencies chosen.

In the multistate model, we modelled risk progression as snapshots in time allowing both forward and backward transitions between the risk states over time. We treated major cardiovascular events as an absorbing state (no progression estimated thereafter) and death from other causes as a competing absorbing state. A transition intensity matrix derived from the multistate model provided transition probabilities and the average time spent in each state. In multistate models, we used the quasi Newton Broyden–Fletcher–Goldfarb–Shanno algorithm for optimization and achieved the 1e-16 convergence criteria. The R code for our final multistate model that provided the transition intensities was

```
final.msm <- msm( state ~ time, subject=ID, data = WHII,  qmatrix = Q.crude, deathexact = c(6, 7), censor = c(8), censor.states = list(c(1,2,3,4,5)), control = list(fnscale = 65000, maxit = 1000, reltol = 1e-16, trace=1, REPORT=1))
```

final.msm: the final msm model
msm: calls the msm command
state: includes low (0-2·49%), intermediate-low (2·5-4·99%), intermediate-high (5-7·49%), high (7·5-14·99%), very high risk (≥15%), major CV event, death and censored states.
time: Follow-up time
subject: unique number that identifies participants
Data: dataset used
qmatrix: initial matrix mentioned earlier
deathexact: exactly observed absorbing states that end the follow-up (major CV event and death).
censor: censoring indicator
censor.states: lists the possible states that participants can be in at the end of follow-up if they do not have major CV event or die.
control fnscale: scaling factor that is approximately equal to -2 × log-likelihood
maxit: maximum number of iterations
reltol: convergence criteria for optimization
trace: calls the msm to show intermediate steps of optimization in order to monitor optimization process
REPORT: Allows following of iterations

Assumptions in Step 4

- The assumption in the multistate model is “the Markov assumption” which assumes “that future evolution only depends on the current state.” We realize that this may not necessarily be the case among individuals who transfer backward from the high-risk category because of initiation of preventive medication and then transfer rapidly back to the high-risk category because of quitting their medication. However, when deriving the screening intervals, we did not allow this backward transition from the high-risk category, thus ensuring no violation to the Markov assumption. Additionally, initiation of primary prevention medication is not common at low and intermediate levels, indicating that the Markov assumption was reasonable at these categories. This was further tested by plotting the observed and estimated proportion of individuals in each state that confirmed that our model fitted the data well.

eFigure 4. Observed and estimated revised ASCVD score prevalence in each phase during follow-up. Blue line indicates the observed, and red dashed line the expected percentages. Time as months on the x-axis, prevalence of each category on the y-axis.

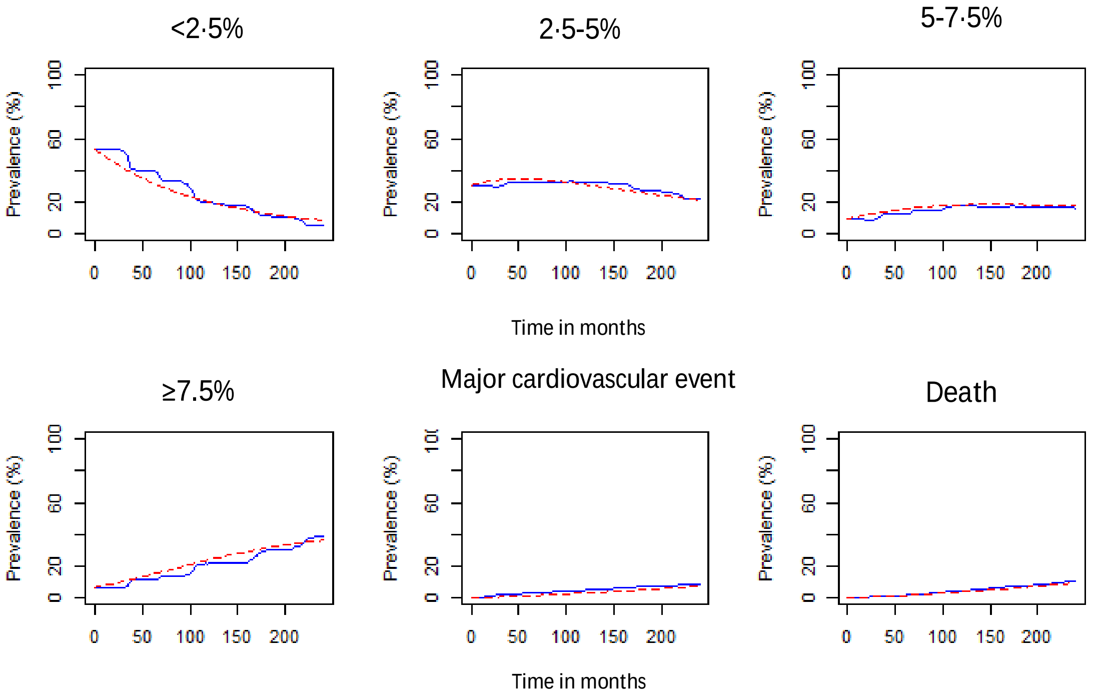

- In addition, an analysis in which we relaxed the Markov assumption did not converge supporting theory that the Markov assumption was reasonable.[2]
- We also assumed that our measurement times were noninformative, that is, the state that individuals occupy does not alter their measurement time. We believe that this is the case, because the measurement intervals in the Whitehall II study were predefined and fixed and thus did not relate to risk categories.

STEP 5

The final msm model provided transition intensities that allowed calculation of probabilities of occupying each state during follow-up. These were calculated with pmatrix.msm command of the msm package and were then used to form equations describing probability of occupying each state over time (eFigure 5). Integration of these risk-category-specific equations allowed modelling of the person years spent unidentified in the high-risk category. eFigure 6 provides examples of transition probabilities derived from our multistate model, and eFigure 7 describes a 7-4-1 screening framework in which the person years spent unidentified in the high-risk category were calculated. eFigure 8 provides the age distribution in each risk category over the follow-up.

eFigure 5. Cumulative probability of progressing to the high 10-year major cardiovascular event risk category (≥7·5%) over time from low (<2·5%), intermediate-low (2·5% to <5%), and intermediate-high (5% to <7·5%) risk categories. AHA/ACC guidelines advise primary prevention for those with high 10-year risk (≥7·5%).

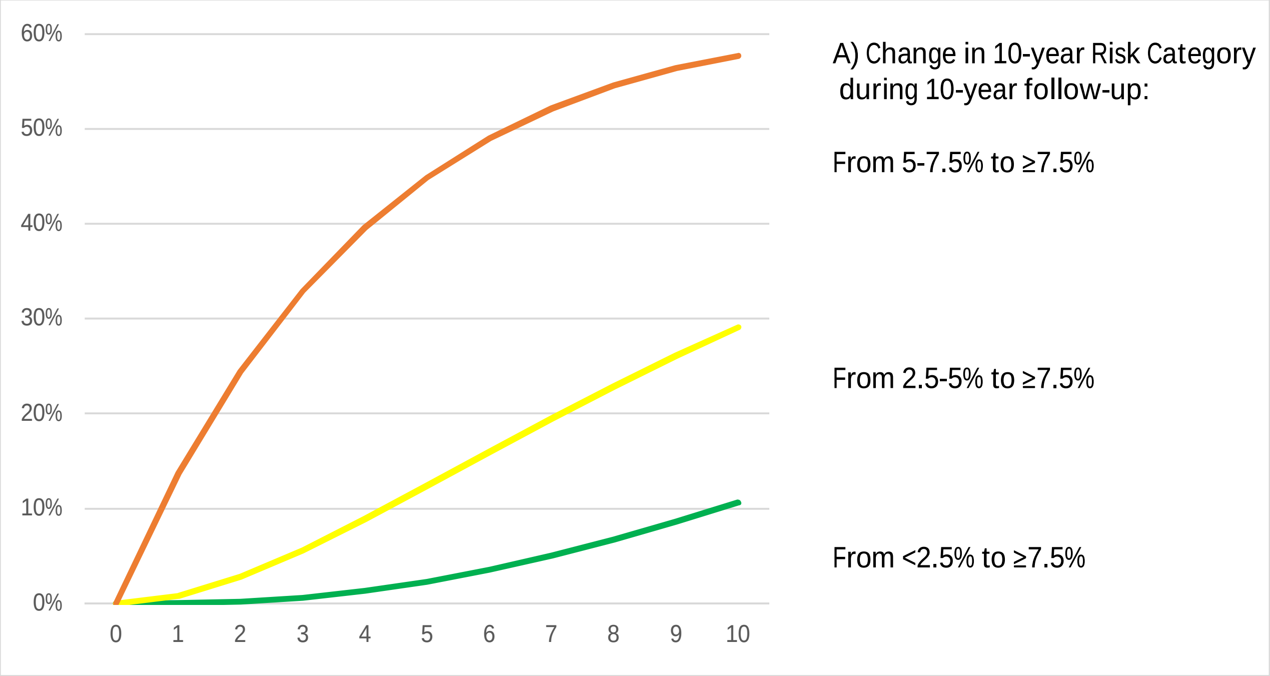

eFigure 6 Estimated probability of progression (%) from low (<2·5%), intermediate-low (2·5% to <5%), intermediate-high (5% to <7·5%), and high (≥7·5%) 10-year major cardiovascular-event-risk categories to other categories in the different follow-up periods. Yellow cells show the percentage of individuals whose risk category did not change, red cells percentage with progressive risk or major cardiovascular events, green cells those at reduced risk. White cells show those who died from non-cardiovascular causes. MACE refers to major (adverse) cardiovascular events. In this model, contrary to figure 7, backward transfers were allowed in order to demonstrate how participants´ risk evolves during follow-up.

A) During 10 years

From category	To category: <2·5%	2·5-5%	5-7·5%	≥7·5%	MACE	Death
<2·5%	34·4	35·7	15·0	10·9	1·9	2·1
2·5-5%	4·5	34·7	24·1	28·5	4·2	3·9
5-7·5%	1·0	12·1	20·9	51·8	7·5	6·8
≥7·5%	0·2	3·4	12·4	62·5	10·6	11·0

B) During 5 years

From category	To category: 0-2·5%	2·5-5%	5-7·5%	≥7·5%	MACE	Death
0-2·5%	57·6	30·9	7·6	2·6	0·6	0·8
2·5-5%	3·9	55·0	24·6	13·3	1·7	1·6
5-7·5%	0·5	12·4	36·0	44·9	3·4	2·8
≥7·5%	<0·1	1·6	10·7	75·8	5·8	6·0

C) During 1 year

From category	To category: 0-2·5%	2·5-5%	5-7·5%	≥7·5%	MACE	Death
0-2·5%	89·3	9·9	0·6	<0·1	0·1	0·1
2·5-5%	1·3	87·4	9·8	0·9	0·3	0·3
5-7·5%	<0·1	4·9	78·6	15·6	0·5	0·3
≥7·5%	<0·1	0·1	3·7	93·6	1·3	1·3

eFigure 7. The 7-, 4-, and 1-year measurement protocol that is based on risk-category-specific transition probabilities for those initially at low risk. Those individuals managing to reduce their risk during follow-up remain in the screening protocol of the highest observed-risk category.*

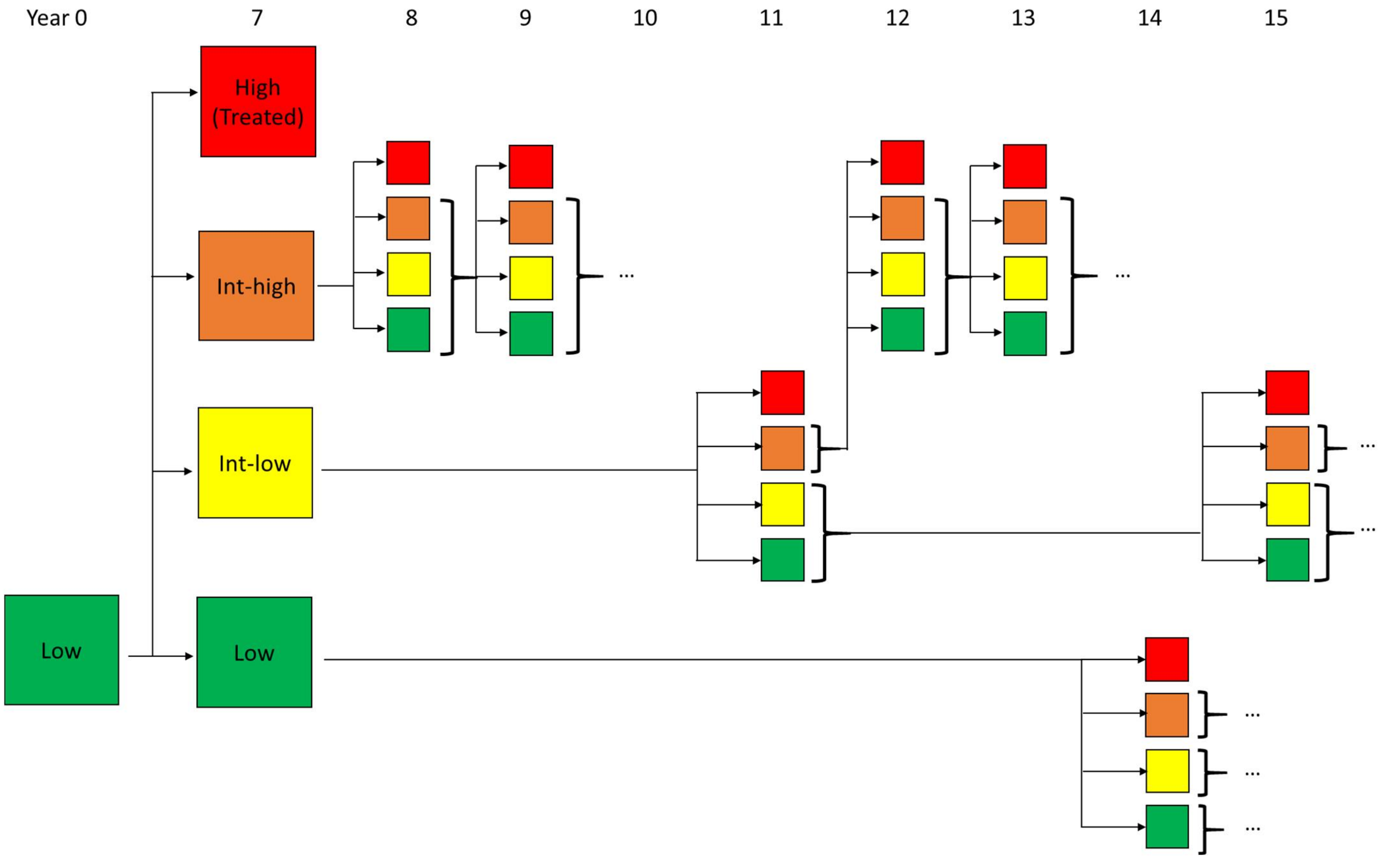

*The additional 49 major cardiovascular events prevented with a 7-4-1 protocol when compared to a uniform 5-year-interval protocol would have increased the proportion of major cardiovascular events originating in the high-risk category from 58·3% to 66·2%, and have decreased the proportion from low- and intermediate-risk categories from 41·7% to 33·8%.

eFigure 8. Age distribution in each 10-year major cardiovascular event-risk category during follow-up. The categories have wider age ranges than the baseline age range because each participant can contribute to more than one risk category, and because risk is recalculated at each measurement over the follow-up.

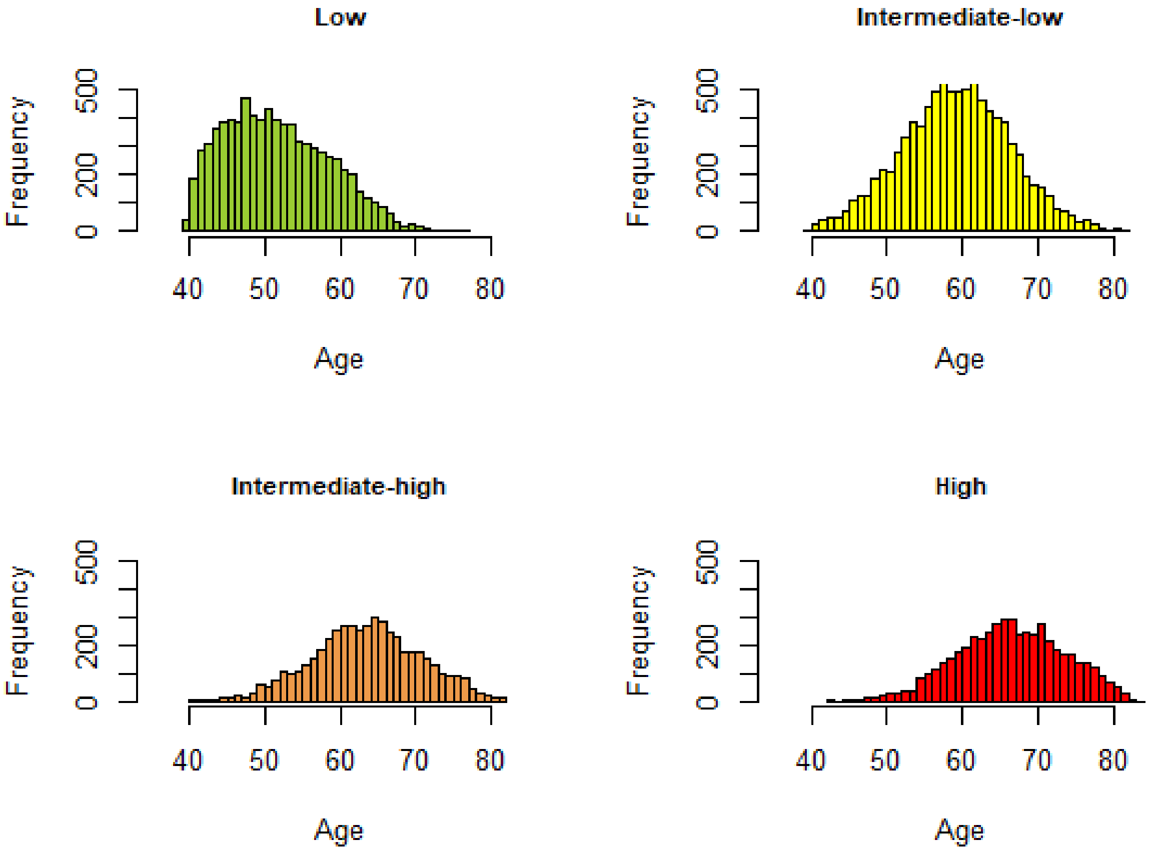

Step 6

Based on the screening-interval-specific person-years spent unrecognized in the high-risk state, we then estimated major cardiovascular events and QALYs saved by means of screening strategies. In these calculations we used estimates by Collins et al.[3] showing that successful statin treatment would prevent 0·01 major cardiovascular events and cause 0·0015, 0·00015, and 0·0001 new diabetes, haemorrhagic stroke, and myopathy cases per person-year. We then multiplied these numbers by the person-years spent unrecognized in the high-risk group with each screening strategy to estimate the major cardiovascular events prevented and new diabetes, haemorrhagic stroke, and myopathy cases caused in Tables 2 and 3 of our paper.
The QALYs were estimated using the West of Scotland Coronary Prevention Study (WOSCOPS) suggesting that successful statin treatment would save 0·00906 QALYs per person-year.[4] This number was also multiplied with the person-years spent unrecognized in the high-risk group with each screening strategy to estimate the QALYs in Tables 2 and 3. Health- check costs in Tables 2 and 3 were derived from a study by Kypridemos et al.[5] and these were multiplied by the number of screenings performed in each screening protocol to estimate screening costs. Estimates of potential benefits of statin treatment derived from the WOSCOPS take into account low compliance. Estimates of QALYs and MACEs include an annual discount factor of 3.5% as recommended by the UK treasury.[6] However, in order to provide conservative estimates, we chose not to discount screening costs.

Assumptions in Step 6

- Our calculations assume a similar compliance in the general population as in WOSCOPS. However, the compliance in randomized trials may be higher than in the general population, due to run-in periods. This suggests that our findings may overestimate the benefits of statin therapy.
- We did not assume a 3.5% annual discount for screening costs, as this may not hold in the future given the rapid development of more cost-effective omics-based health checks. This suggests that our cost estimates for risk-category-specific screening intervals are likely to be conservative.

Step 7

We studied factors driving the risk towards the high-risk state in the Cox proportional hazards model using incident high-risk state as the outcome. This model included the following variables: baseline age, baseline 10-year major cardiovascular-event risk, sex, total cholesterol, high-density lipoprotein, systolic blood pressure, use of antihypertensive medication (yes/no), smoking (yes/no), diabetes (yes/no), socioeconomic status (low/intermediate/high), ethnicity (white/non-white), general health questionnaire (cut-off at 5≤), family history of major cardiovascular events (stroke or myocardial infarction in either parent or in any sibling), and marital status (married, cohabiting, single, divorced, or widowed). This analysis showed that the only factors included in the revised ASCVD score were associated with risk for reaching the high-risk category. The strongest associations were for smoking, diabetes, and systolic blood pressure.

Hazard ratios and 95% confidence intervals for reaching the high-risk state per standard deviation (continuous variables) or for unit increase (binary variables):

Age:	1.37 (1.30-1.45)
Total cholesterol:	1.24 (1.20-1.29)
High-density lipoprotein:	0.70 (0.67-0.73)
Systolic blood pressure:	1.65 (1.57-1.72)

Binary variables

Female sex:	0.73 (0.67-0.80)
Antihypertensive medication:	1.49 (1.25-1.77)
Smoking:	2.80 (2.46-3.18)
Diabetes:	1.85 (1.39-2.48)

We also explored which risk factors changed in parallel with risk progression to, and risk reduction from the high-risk category. We first identified individuals at intermediate or low risk at the first screening and at high risk at the third screening, and compared the values of their risk factors at the first and third screenings. eTable 1 A shows the baseline risk factors in individuals when in the low or intermediate risk category, and eTable 1 B shows risk factors among the same individuals when at high risk in the third screening.

eTable 1. Risk factors for individuals in the low- or intermediate-risk category at baseline (Part A) and in the high-risk category ten years later (Part B).

Part A

Variable	Participants	Mean	Std. Dev.
Total cholesterol (mmol/l)	1150	6.73	1.09
High-density lipoprotein (mmol/l)	1150	1.31	0.36
Proportion of smokers	1150	0.15	0.36
Age in years	1150	53.04	5.52
Proportion of diabetes	1150	0.02	0.15
Proportion on antihypertensive medication	1150	0.07	0.25
Systolic blood pressure (mmHg)	1150	127.08	12.88

Part B

Variable	Participants	Mean	Std. Dev.
Total cholesterol (mmol/l)	1150	5.83	1.10
High-density lipoprotein (mmol/l)	1150	1.40	0.37
Proportion of smokers	1150	0.13	0.34
Age in years	1150	64.40	5.49
Proportion of diabetes	1150	0.17	0.38
Proportion on antihypertensive medication	1150	0.59	0.49
Proportion on lipid lowering medication	1150	0.16	0.37
Systolic blood pressure (mmHg)	1150	140.30	16.62

eTables 1 A and B show that participants progressed to higher risk despite having reduced cholesterol and increased high-density lipoprotein. The change in these values partly reflects the increasing use of lipid-lowering medications (use of lipid-lowering medication was 0% at baseline because it was not widely available in 1991/93). The main factors that increased and led to high risk were increase in systolic blood pressure, increasing use of antihypertensive medication, higher rates of diabetes, and increasing age.

We also compared the risk factors at the third and fifth screenings in those individuals who managed to reduce their risk from high to intermediate or low risk between these screenings. eTable 2 A describes the risk factors at the third and eTable 2B at the fifth screening.

eTable 2. Risk factors for individuals in the high-risk category (Part A) and in the intermediate- or low-risk category ten years later (Part B).

Part A

Variable	Participants	Mean	Std. Dev.
Total cholesterol (mmol/l)	93	6.18	1.13
High-density lipoprotein (mmol/l)	93	1.39	0.40
Proportion of smokers	93	0.24	0.43
Age in years	93	61.46	5.27
Proportion of diabetes	93	0.11	0.31
Proportion on antihypertensive medication	93	0.56	0.50
Proportion on lipid-lowering medication	93	0.06	0.25
Systolic blood pressure (mmHg)	93	136.28	15.18

Part B

Variable	Participants	Mean	Std. Dev.
Total cholesterol (mmol/l)	93	4.34	0.94
High-density lipoprotein (mmol/l)	93	1.56	0.40
Proportion of smokers	93	0.02	0.15
Age in years	93	70.62	5.27
Proportion of diabetes	93	0.11	0.31
Proportion on antihypertensive medication	93	0.53	0.50
Proportion on lipid-lowering medication	93	0.77	0.42
Systolic blood pressure (mmHg)	93	120.38	15.56

This table shows that risk reduction was driven by a dramatic drop in smoking prevalence from 24% to 2% as well as decreases in total cholesterol, in high-density lipoprotein, and in systolic blood pressure.

STEP 8

To explore whether selection bias might have occurred due to missing data, we undertook sensitivity analysis in which we used multiple imputation with chained equations based on the Nelson-Aalen estimator, on outcome data (major cardiovascular event and death), and on repeated measurements of (observed) 10-year risk, socio-economic status, alcohol consumption, physical activity, body mass index, ethnicity (white or non-white), DASH-diet score, family history of myocardial infarction or stroke (in either parent or any sibling), and a general-health questionnaire (with 30 questions) (N= 10 109).

We imputed the data in wide form to take into account the clustering of repeated measurements within individuals. The diagnostics of our imputation model suggested that 10 iterations and 25 imputations were sufficient to produce reproducible results. The 25 datasets produced from imputations were then analysed separately with the multistate Markov models, and we combined the results using Rubin's rules. This procedure takes into account the uncertainty in the imputation as well as uncertainty due to random variation. In these analyses, we examined whether the association of the revised ASCVD-estimated risk categories with incident major cardiovascular events in the imputed datasets (that aims to control the potential selection bias) differed from that association in our main analysis that included missing data. eFigures 9 A-C below show the similarity in the cumulative probability of reaching the high-risk category in complete-case analysis and after multiple imputation.

eFigure 9A. Cumulative probability of progressing to the high 10-year major cardiovascular event-risk category (≥7·5%) over time from the low (<2·5%) risk category. AHA/ACC guidelines advise primary prevention for those at high 10-year risk (≥7·5%). Blue bars describe complete case analysis and red bars analysis with 25 imputed dataset.

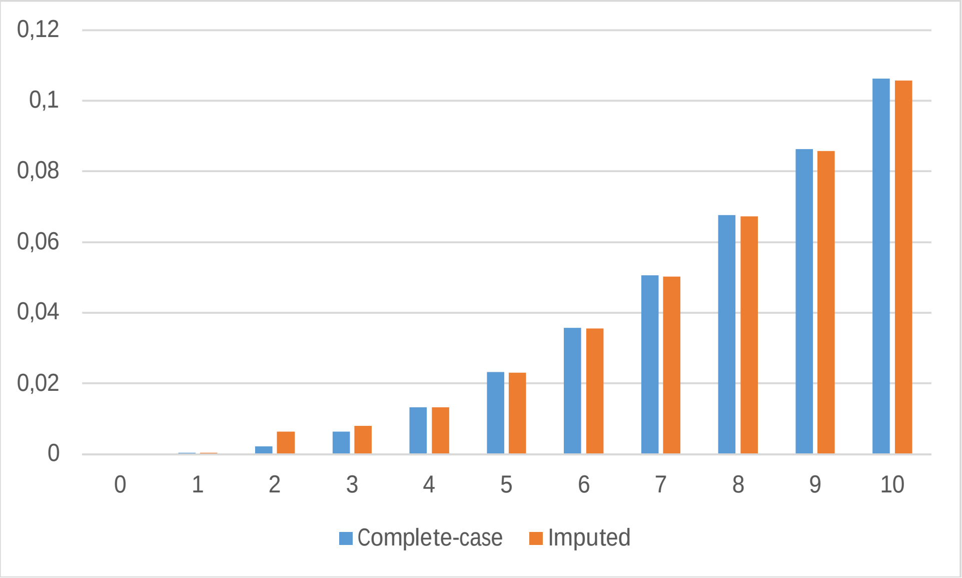

eFigure 9B. Cumulative probability of progressing to the high 10-year major cardiovascular event-risk category (≥7·5%) over time from the intermediate-low (2·5% to <5%) risk category. AHA/ACC guidelines advise primary prevention for those at high 10-year risk (≥7·5%). Blue bars describe complete case analysis and red bars analysis with 25 imputed dataset.

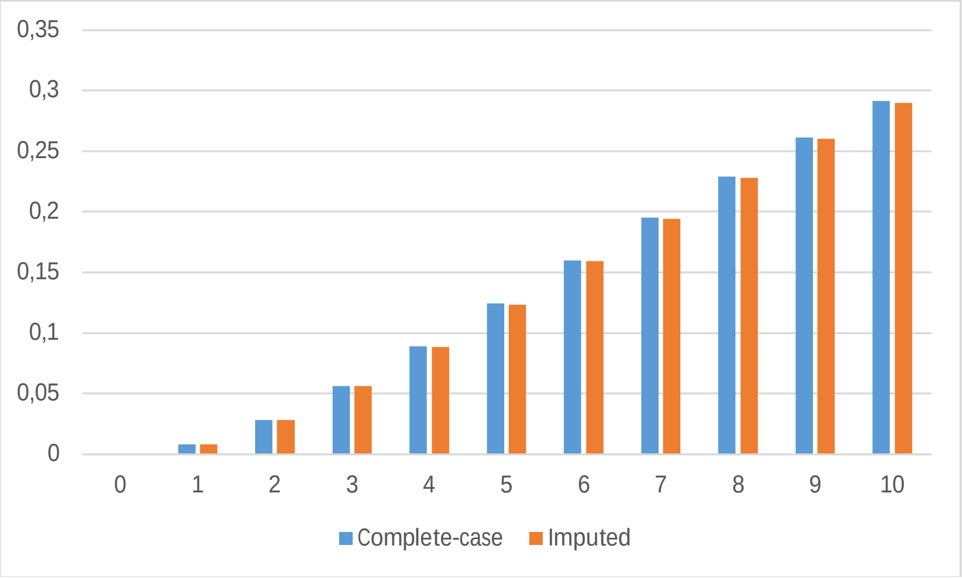

eFigure 9C. Cumulative probability of progressing to the high 10-year major cardiovascular event- risk category (≥7·5%) over time from the intermediate-high (5% to <7·5%) risk category. AHA/ACC guidelines advise primary prevention for those at high 10-year risk (≥7·5%). Blue bars describe complete case analysis and red bars analysis with 25 imputed dataset.

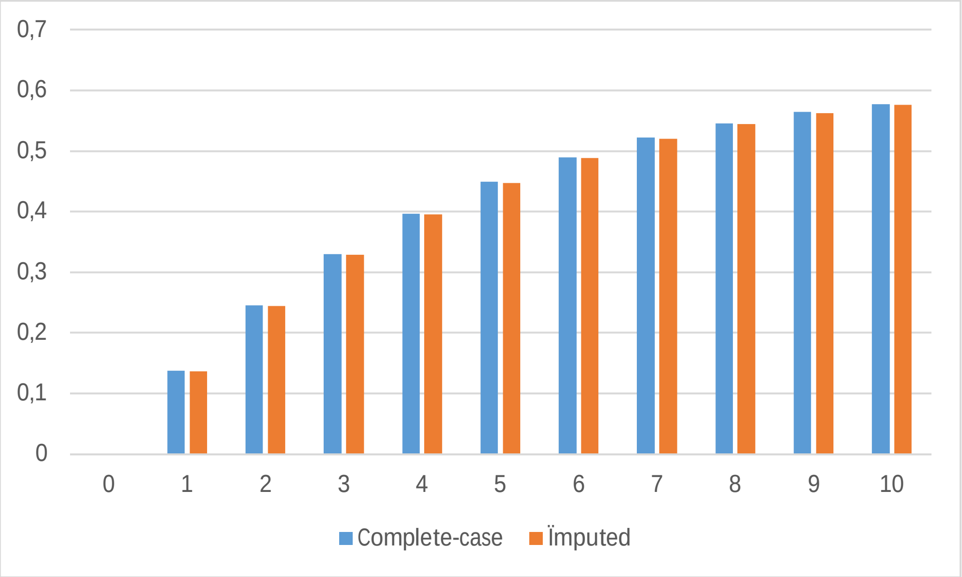

References

1. Goff DC,Jr, Lloyd-Jones DM, Bennett G, et al. 2013 ACC/AHA guideline on the assessment of cardiovascular risk: a report of the American College of Cardiology/American Heart Association Task Force on Practice Guidelines. *Circulation* 2014; **129**(25 Suppl 2): S49-73. doi:10.1161/01.cir.0000437741.48606.98 [doi].

2. Jackson C. R statistical software programme msm: Multi-State Markov and Hidden Markov Models in Continuous Time. https://rdrr.io/cran/msm/man/msm.html (accessed in February 6, 2019).

3. Collins R, Reith C, Emberson J, et al. Interpretation of the evidence for the efficacy and safety of statin therapy. *Lancet* 2016; **388**(10059): 2532-61. doi:S0140-6736(16)31357-5 [pii].

4. McConnachie A, Walker A, Robertson M, et al. Long-term impact on healthcare resource utilization of statin treatment, and its cost effectiveness in the primary prevention of cardiovascular disease: a record linkage study. *Eur Heart J* 2014; **35**(5): 290-8. doi:10.1093/eurheartj/eht232 [doi].

5. Kypridemos C, Collins B, McHale P, Bromley H, Parvulescu P, Capewell S, O'Flaherty M. Future cost-effectiveness and equity of the NHS Health Check cardiovascular disease prevention programme: Microsimulation modelling using data from Liverpool, UK. *PLoS Med* 2018; **15**(5): e1002573. doi:10.1371/journal.pmed.1002573 [doi].

6. Lowe J. Intergenerational wealth transfers and social discounting: Supplementary Green Book guidance. *HM Treasury* 2008; Available from: https://www.gov.uk/government/uploads/system/uploads/attachment_data/file/193938/Green_Book_supplementary_guidance_intergenerational_wealth_transfers_and_social_discounting.pdf.
